# Retroperitoneal Spindle Cell Rhabdomyosarcoma With Compression Features in a 48-Year-Old Male: A Case Report

**DOI:** 10.7759/cureus.29622

**Published:** 2022-09-26

**Authors:** Krishna Ramesh, Anzal Jangda, Swetha Annam, Marium A Mangrio, Krishna Sajeev, Anish Kumar, Mahrukh Qayyum, Momal Jamali, Allahyar Yahya, Hummaz Mehbub

**Affiliations:** 1 Internal Medicine, Ramaiah Medical College, Bangalore, IND; 2 Internal Medicine, Ziauddin University, Karachi, PAK; 3 Pulmonary Medicine, Osmania Medical College, Hyderabad, IND; 4 Internal Medicine, Bhaskar Medical College, Hyderabad, IND; 5 Medicine and Surgery, Sukkur Civil Hospital, Sukkur, PAK; 6 Internal Medicine, Government Medical College Kannur, Pariyaram, IND; 7 Internal Medicine, Ghulam Muhammad Mahar Medical College Hospital, Sukkur, PAK; 8 Internal Medicine, Allama Iqbal Medical College, Lahore, PAK; 9 Internal Medicine, Dow University of Health Sciences, Karachi, PAK; 10 General Surgery, Akhtar Saeed Medical & Dental College, Lahore, PAK

**Keywords:** embryonal rhabdomyosarcoma, retroperitoneal mass, spindle cell rhabdomyosarcoma, p-rms, rhabdomyosarcoma (rms), rms

## Abstract

Spindle cell rhabdomyosarcoma (SC-RMS) is an unprecedented version of embryonal rhabdomyosarcomas (RMSs) that emerges from the mesenchyme with the capacity to differentiate into skeletal muscle cells. Retroperitoneal RMS is extremely rare in the adult population. We present the case of a primary spindle cell retroperitoneal RMS with compression features. Investigation-based diagnosis of RMS is difficult due to the lack of specificity of clinical findings. Radiology does not help in making an accurate diagnosis. Surgical removal of the tumor followed by chemotherapy and radiotherapy is the best possible treatment for RMS in adults. SC-RMS has a poor long-term prognosis. To our knowledge, such cases of retroperitoneal SC-RMS compressing the abdominal viscera and resulting in hydroureteronephrosis have never been reported before.

## Introduction

Soft-tissue sarcomas arising from the rhabdoid muscle are referred to as rhabdomyosarcoma (RMS); however, almost 47% of the RMSs do not originate from these muscles. Retroperitoneal RMS is uncommon in the adult population [1]; consequently, knowledge about its medical findings and the pathological process is not sufficiently available. Due to a lack of literature, limited information about its diagnosis and management is currently available [2]. The clinical course of adult RMS is more aggressive than the cases occurring in the pediatric population. Although embryonal RMS is rare in the adult population, the spindle cell variant is considered an infrequent type of embryonal RMS (only 3% of all RMS cases in the Intergroup Rhabdomyosarcoma Study) [3].

Spindle cell RMS (SC-RMS) makes up <10% of adult rhabdomyosarcomas and the most common site is the head and neck; however, it is found at variant locations, such as the diaphragm, paratesticular region, and uterus. Rare anatomical sites include the solid organs and retroperito­neum, and only three cases of retroperito­neal rhabdomyosarcomas have formerly been presented [1,4]. To our knowledge, we report the first ever case of sclerosing SC-RMS emerging within the retroperitoneal area in a 48-year-old male extending into the descending mesocolon and compressing the left ureter, left common iliac artery, and urinary bladder and causing hydroureter and hydronephrosis.

## Case presentation

A 48-year-old male patient presented to the hospital’s surgical outpatient department (OPD) complaining of pain in the abdomen and lower back. Detailed history revealed that the patient was having dull and aching pain in the umbilical region and lower back for two months. The pain was mild in intensity but aggravated on bending and lifting weights. It was however relieved with the use of over-the-counter non-steroidal anti-inflammatory drugs.

**Figure 1:**
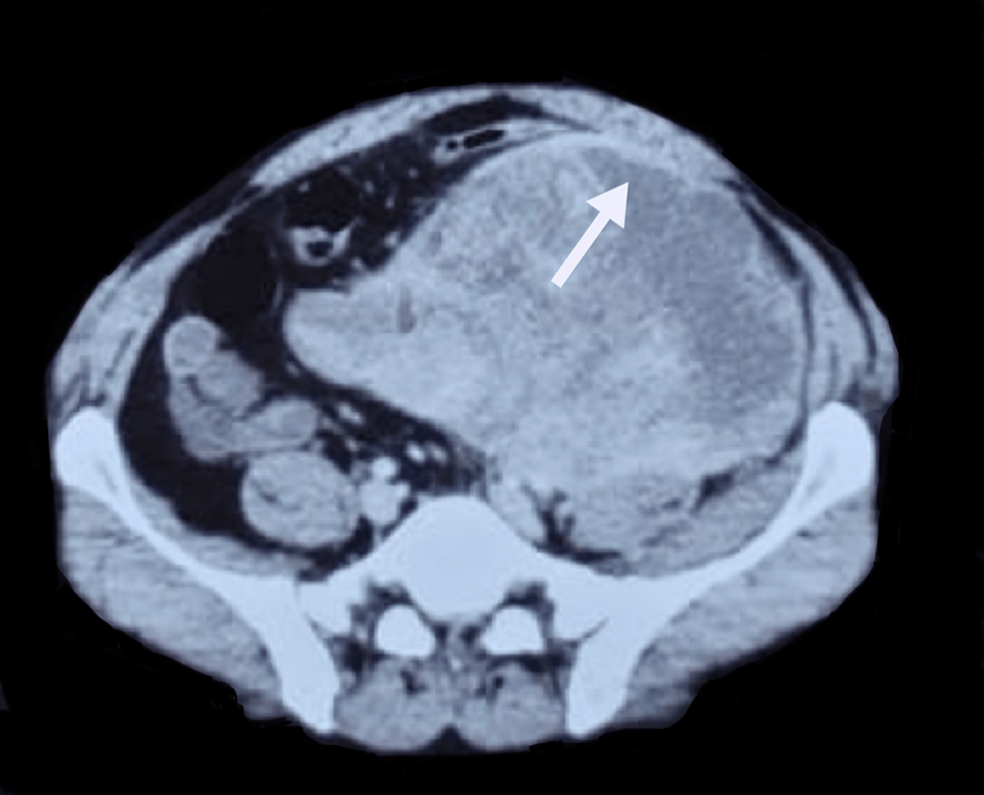
CT scan showing the tumor mass originating from iliopsoas muscle with patchy contrast enhancement of the mass.

**Figure 2:**
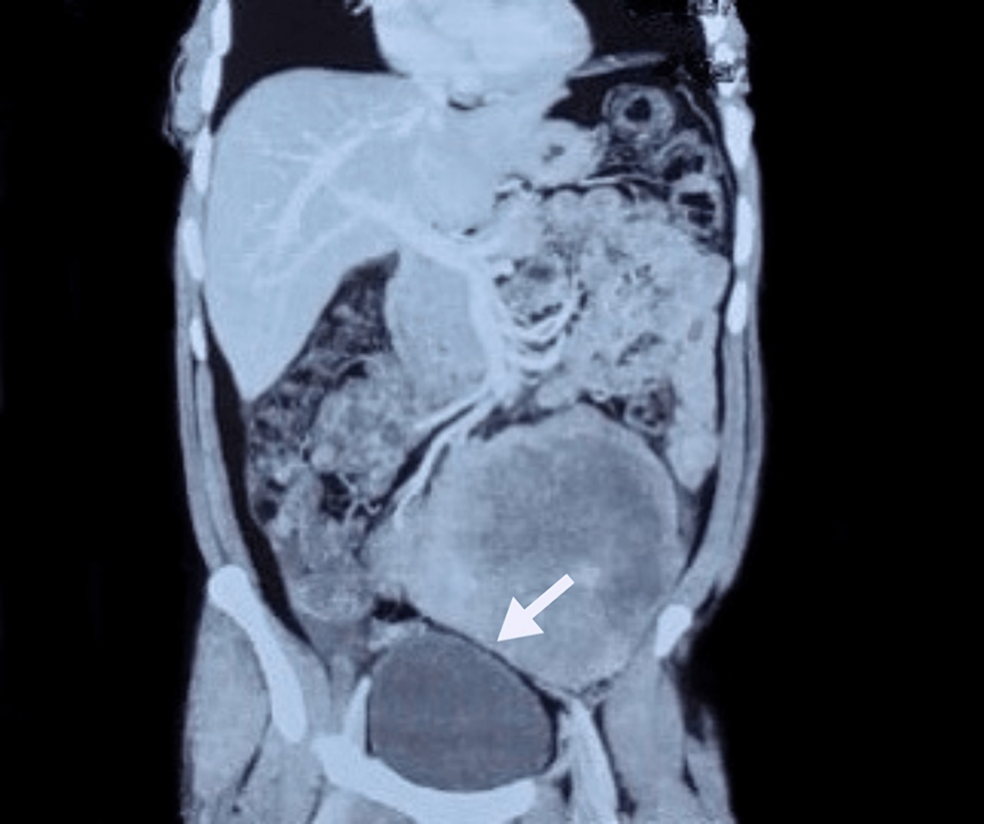
Coronal section of CT scan shows tumor mass compressing the dome of the urinary bladder.

Physical examination of the inguinoscrotal region and abdomen was unremarkable, except for mild left flank tenderness. During his evaluation, an ultrasound of the abdomen and kidney, ureter, and bladder (KUB) was ordered, which revealed a heterogenous soft tissue mass, predominantly hypoechoic measuring 120 X 154 mm and reaching up to a maximum depth of 137 mm in the umbilical region. Left-sided hydronephrosis and hydroureter (maximum diameter of 18.3 mm) were also noted. However, it showed absent vascularity on Doppler. Laboratory investigations were unremarkable. A CT scan of the abdomen and pelvis confirmed the large soft tissue mass measuring 12 X 17 cm, showing patchy post-contrast enhancement and areas of subtle necrosis, and a possibility of neoplastic etiology. CT scan also showed a significant mass effect along the course of the ureter with resultant upstream mild hydroureteronephrosis and a delayed left pyelogram. Small free fluid was seen in the perisplenic and perihepatic regions. However, no evident lymphadenopathy and lytic or sclerotic lesions were seen in the visualized skeleton (Figures 1, 2).

**Figure 3:**
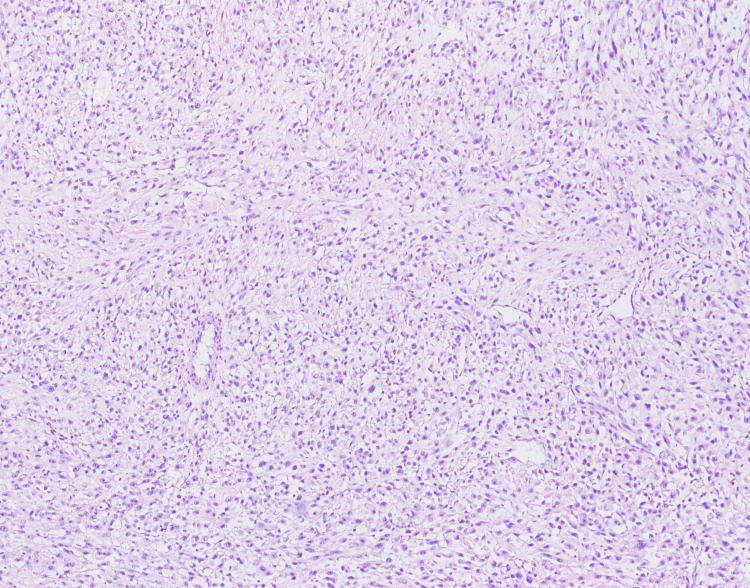
Photomicrograph shows pleomorphic rhabdomyosarcoma with haphazardly spindle cells and scattered cells with hyalinized stroma.

**Figure 4:**
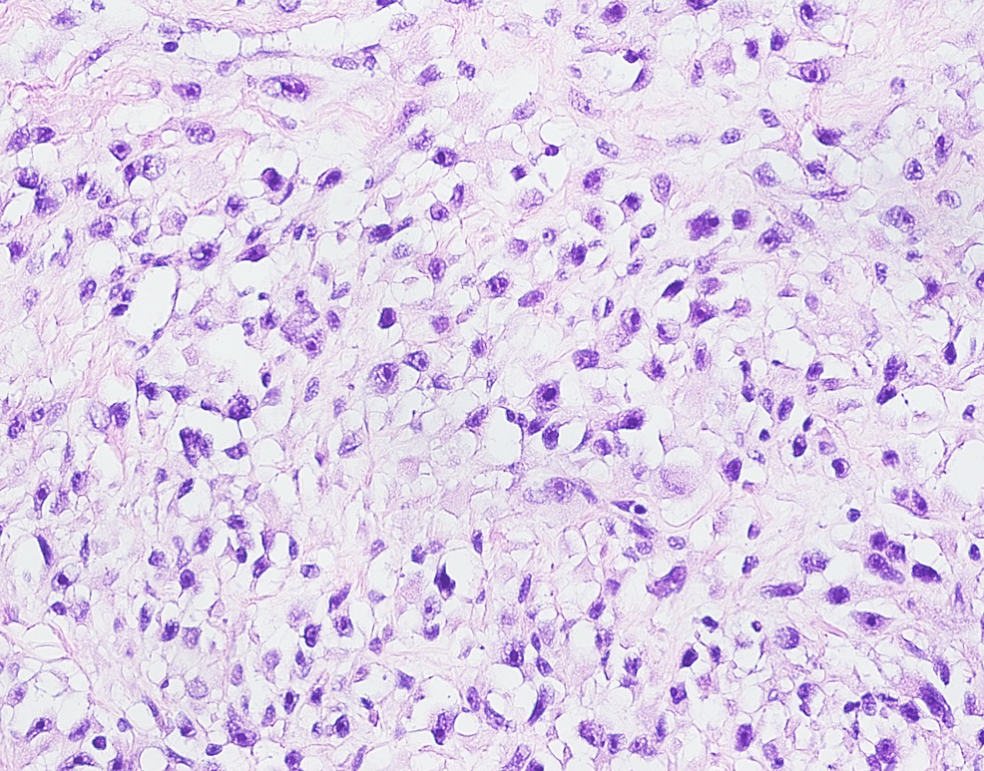
High power view shows neoplastic spindle to round cells with distinct eosinophilic stringy cytoplasm and moderate atypia and mitosis.

**Figure 5:**
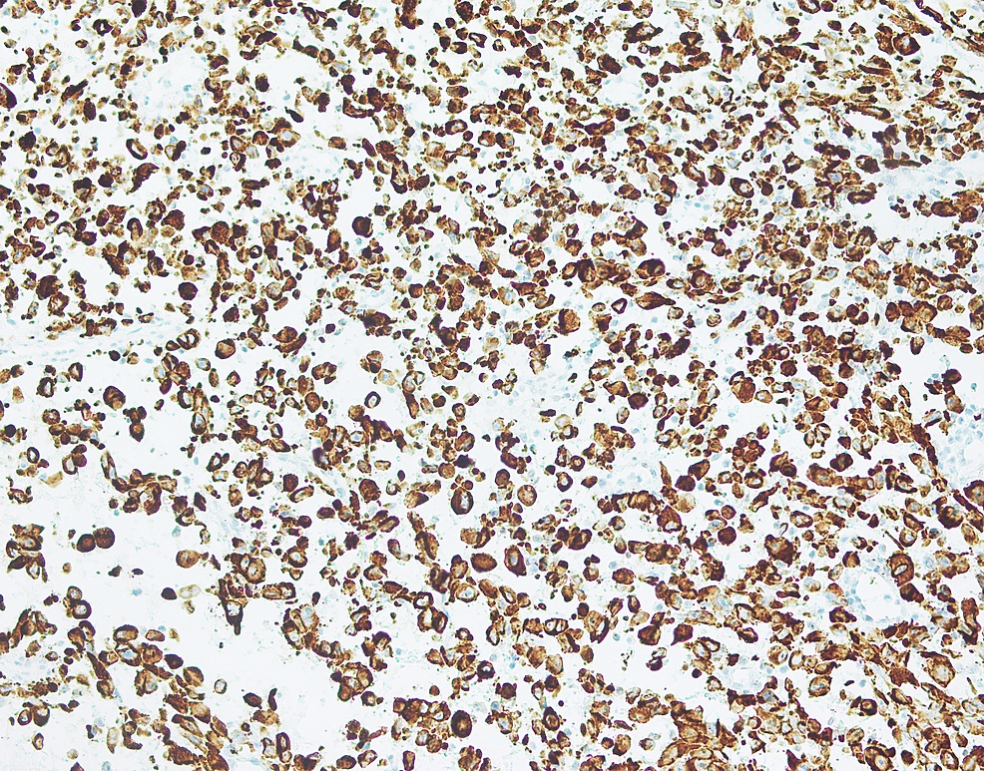
Neoplastic cells show diffuse immunopositivity for desmin.

Concerning the neoplastic etiology, a laparoscopic incisional biopsy was taken which showed a malignant neoplasm composed of atypical and moderately pleomorphic spindle cells with elongated nuclei (Figures 3). Brisk mitotic activity was also observed (Figure 4). Immunohistochemistry showed positivity for desmin (Figure 5) and MyoD1 and negative for SMA, CDX2, myogenin, SOX10, HMB45, CK7, and DOG1. The diagnosis of pleomorphic SC-RMS was made.

The concern for malignancy and compression findings formed an indication for excisional laparotomy. Upon exploration, there was a large soft tissue mass confined to the retroperitoneum below the level of the left kidney extending into the left descending mesocolon and encasing the left ureter along most of the length. The left common iliac artery was also encased by the lower pole of the tumor. No peritoneal deposits were seen. The liver, spleen, bowel loops, and urinary bladder were normal. The tumor mass along with a rim of healthy tissue was completely excised and sent for histopathology. The left ureter, left common iliac artery, and urinary bladder were decompressed.

**Figure 6:**
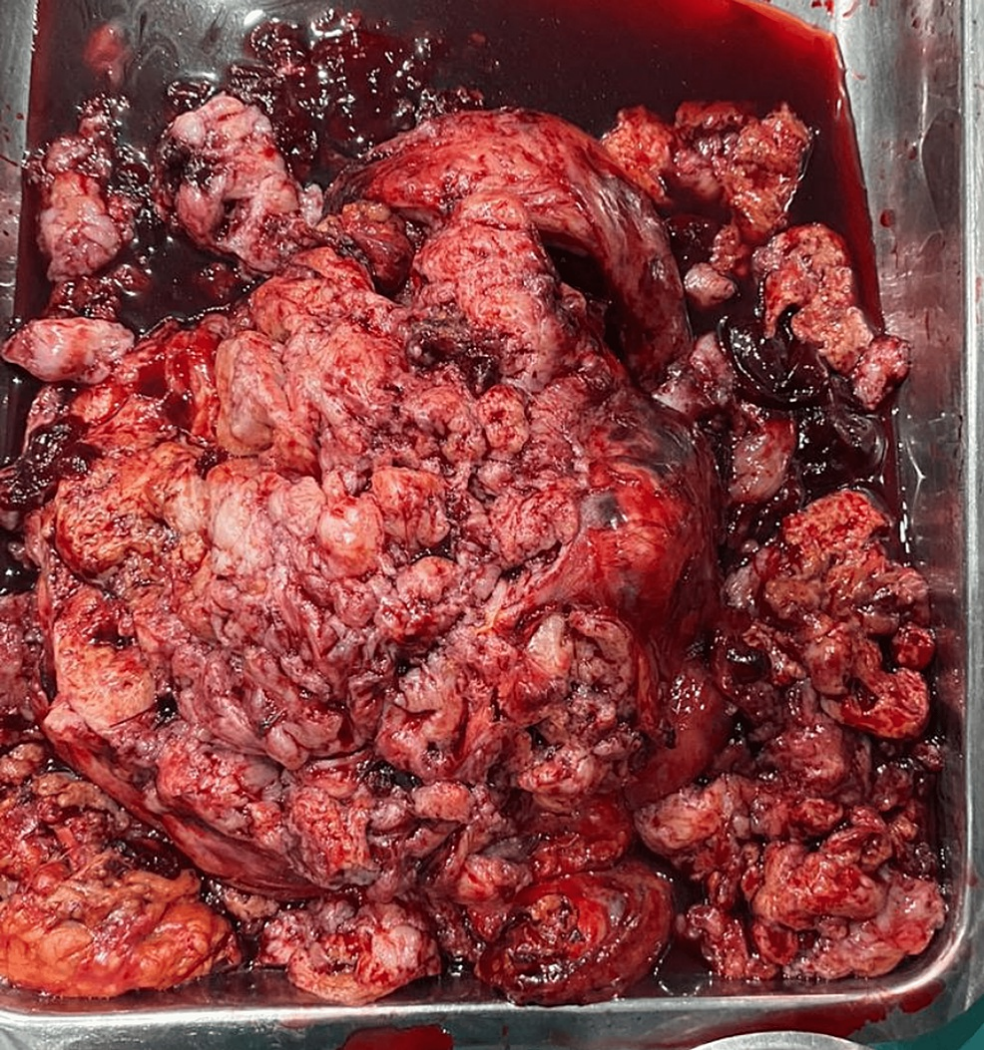
Distorted tumor mass with soft to firm tan-white, multilobulated with extensive hemorrhagic and necrotic areas alongside multiple separate tissue pieces

At pathology, an unoriented, distorted mass measuring 220 X 160 X 80 mm with soft to firm tan-white, multilobulated with extensive hemorrhagic and necrotic areas was seen (Figure 6). Multiple separate tissue pieces collectively measuring 130 X 100 X 70 mm were also recorded.

Histopathology was consistent with the previous findings. Multiple sections showed neoplastic spindled cells arranged in single file arrays in a hyalinized eosinophilic matrix. There was moderate to marked atypia and brisk mitotic activity. Immunohistochemistry was consistent with the previous biopsy (Figures 3, 4, 5). Hence, a definitive diagnosis of pleomorphic sclerosing SC-RMS was confirmed. Postoperatively, the patient showed an unremarkable recovery. He was then referred for chemotherapy and radiotherapy.

## Discussion

RMAs are common in children but rare in adults. The new World Health Organization (WHO) classification divides RMSs into four groups: alveolar RMA, embryonal RMA, spindle cell/sclerosing RMA, and pleomorphic RMA. Adult RMS begins in the major skeletal muscle [5]. It originates from the embryonic mesenchyme and has the potential to differentiate into rhabdoid muscle cells. Nearly half of all the soft tissue sarcomas in children are RMSs compared to only 3% in adults [6]. Retroperitoneal SC-RMS is an exceptionally rare tumor in adults, with only three cases (two of them are male) previously described in the literature [7].

Preoperative diagnosis is challenging due to unclear symptoms. Preoperative imaging contributed little to the final diagnosis, showing a nonspecific mass with patchy enhancement [8]. SC-RMS presents particular problems in making the differential diagnoses in adults and needs to be differentiated from other such malignancies, including leiomyosarcoma, sarcomatoid carcinoma, fibrosarcoma, and malignant nerve sheath tumors with heterogeneous rhabdomyocyte differentiation. Likewise, histology and immunohistochemical staining for skeletal muscle markers help distinguish SC-RMSs from other spindle cell tumors [7].

The size and consistency of RMAs vary widely. These tumors are well-circumscribed but unencapsulated and generally have a tendency to infiltrate widely into adjacent tissues [2]. Sections show a firm white-to-tan mass with a leiomyoma-like or leiomyosarcoma-like appearance, especially when existing in the uterus of an adult female. Necrosis is a common finding of cystic degeneration and scattered areas of hemorrhage and can also be secondary to preoperative embolization. The clinical setting and morphology should trigger appropriate immunohistochemical examination, which helps distinguish this entity from various other spindle cell tumors [3].

Histopathology is dominated by the constant cellular proliferation of elongated spindle cells arranged in fascicles or spirals in a herringbone growth pattern similar to leiomyosarcoma. The nucleus is centrally located, with blunt or fusiform ends, the nucleoli are small to inconspicuous or prominent, and the cytoplasm is eosinophilic. Mitotic figures are easy to understand, including atypical forms [3]. RMS on histology is a hypercellular tumor, primarily composed of bundles of spindle cells, with elongated nuclei, pale pink cytoplasm, and inconspicuous nucleoli, resembling rare fibrosarcomas, leiomyosarcoma seen in adults [7]. Rhabdomyocytosis and muscle-specific markers of vimentin, myogenin, SMA, and desmin were utilized for pathological examination to validate the diagnosis [9,10]. A majority of RMAs indicate elevated levels of markers of muscle cell differentiation, such as myogenin and MyoD1 [5]. SC-RMA constantly reacts with myogenic markers such as titin, troponin D, myoglobin, desmin, and MyoD1 [3].

RMS can be treated in three ways including surgery, radiation therapy to control residual large or small tumors, and systemic combination chemotherapy for primary cytoreduction and eradication of large and micrometastases. Surgery is the fastest way to erode the disease but it should always be performed if subsequent functional or cosmetic damage is not greatly affected. Surgery consists of complete resection of the primary tumor and margins of surrounding uninvolved tissue during the initial surgery and any subsequent surgery. After the initial excision, if the microscopic residual disease is found, the area should be re-excised before any other nonsurgical treatment. Debulking surgery is of no value because neoadjuvant therapy and initial biopsy result in tumor shrinkage, allowing complete resection in a second observational surgery [8,11].

Sultan et al. reported that adults with RMA have a considerably lower prognosis than children (five-year overall survival, 27% vs 61% in children). Adult tumors were more certain to be located at a critical site (65% vs 55% in children) [12]. According to the IRSG protocol, all RMS should receive radiation therapy to achieve long-term local control of the tumor. Once diagnosed or resected, all RMS patients must receive combination chemotherapy. It considerably progresses survival [2]. Disease responsive to chemotherapy is linked with better metastasis-free intervals [13].

## Conclusions

SC-RMS produces vague clinical features, especially when they arise in the retroperitoneum, which leads to advanced disease at the time of presentation. Noninvasive diagnostic modalities including radiology play little role in making a final diagnosis. Histopathology, however invasive, can be conclusive. A high degree of suspicion remains a significant factor in the diagnosis of such rare tumors. Treatment includes a three-prong strategy with resection of the tumor followed by chemotherapy and radiotherapy.

## References

[1] Nascimento AF, Fletcher CD (2005). Spindle cell rhabdomyosarcoma in adults. Am J Surg Pathol.

[2] Yadav SK, Sinha DK, Ahmed A, Azhar T, Sinha M (2015). Primary intra-abdominal rhabdomyosarcoma in an adult: an unusual presentation and review of literature. Indian J Surg Oncol.

[3] Carroll SJ, Nodit L (2013). Spindle cell rhabdomyosarcoma: a brief diagnostic review and differential diagnosis. Arch Pathol Lab Med.

[4] Fernando Val-Bernal J, Fernández N, Gómez-Román JJ (2000). Spindle cell rhabdomyosarco­ma in adults. A case report and literature re­view. Pathol Res Pract.

[5] Allameh F, Karkan MF, Rakhshan A (2017). Renal rhabdomyosarcoma in a young woman with gross hematuria: a case report. Int J Cancer Manag.

[6] Furlong MA, Mentzel T, Fanburg-Smith JC (2001). Pleomorphic rhabdomyosarcoma in adults: a clinicopathologic study of 38 cases with emphasis on morphologic variants and recent skeletal muscle-specific markers. Mod Pathol.

[7] Yu L, Yang SJ (2014). Spindle cell rhabdomyosarcoma of the retroperitoneum: an unusual case developed in a pregnant woman but obscured by pregnancy. Int J Clin Exp Pathol.

[8] Shirafkan Md A, Boroumand Md N, Komak Md S, Duchini Md A, Cicalese Md L (2015). Pancreatic pleomorphic rhabdomyosarcoma. Int J Surg Case Rep.

[9] Mendenhall WM, Indelicato DJ, Scarborough MT (2009). The management of adult soft tissue sarcomas. Am J Clin Oncol.

[10] Little DJ, Ballo MT, Zagars GK (2002). Adult rhabdomyosarcoma: outcome following multimodality treatment. Cancer.

[11] Agarwala S (2006). Paediatric rhabdomyosarcomas and nonrhabdomyosarcoma soft tissue sarcomas. J Indian Assoc Pediatr Surg.

[12] Sultan I, Qaddoumi I, Yaser S, Rodriguez-Galindo C, Ferrari A (2009). Comparing adult and pediatric rhabdomyosarcoma in the surveillance, epidemiology and end results program, 1973 to 2005: an analysis of 2,600 patients. J Clin Oncol.

[13] Maurer HM, Gehan EA, Beltangady M (1993). The Intergroup Rhabdomyosarcoma Study-II. Cancer.

